# ANEST narrative–affect dataset (ANAD v1): A large-scale derived feature resource for quantifying narrative–affective discrepancy

**DOI:** 10.1016/j.dib.2026.112643

**Published:** 2026-03-04

**Authors:** Ryan SangBaek Kim

**Affiliations:** Ryan Research Institute (RRI), 4 Rue Saint-Bernard, 75011 Paris, France

**Keywords:** Narrative-affect discrepancy, NADI, Affective computing, Narrative complexity, VADER sentiment, Derived feature dataset, Computational psychology, Emotion AI

## Abstract

Narrative psychology, affective science, and computational social science all assume that how people tell stories about their lives is deeply intertwined with how they feel. Yet most empirical work either treats narrative structure and affective valence as separate constructs, or focuses on one dimension while ignoring the other. Here I present the ANEST Narrative–Affect Dataset (ANAD v1), a large-scale derived feature resource based on *N* = 351,734 human-written, English-language narratives drawn from public online discussions of romantic and relational life. Each observation is represented by three layers of derived annotation: (i) basic structural descriptors, (ii) a language-based index of narrative complexity (Level of Complexity; LoC), and (iii) a normalized affective polarity score derived from a rule-based sentiment model. From these components, I define the Narrative–Affect Discrepancy Index (NADI), which quantifies the gap between narrative complexity and expressed affect on a common 0–10 scale. NADI is offered as a computable, operational indicator rather than a validated psychological construct. The dataset is openly available via Zenodo (https://doi.org/10.5281/zenodo.18680687). No verbatim text is redistributed; the released files contain only derived, non-identifiable feature representations. This data article outlines the collection pipeline, scoring procedures, core distributions, and recommended use cases.

Specifications Table

**Table:** 

Subject	Social Science
Specific subject area	Computational narrative psychology and affective computing
Type of data	Table (Parquet, CSV); Processed; Filtered
Data collection	351,734 English-language personal narratives collected from Reddit r/relationships (2012–2023) via Pushshift API [1] and official Reddit API. Each text annotated with structural descriptors, Level of Complexity (LoC) via heuristic discourse-marker pipeline, VADER sentiment (compound score rescaled to 0–10), and Narrative–Affect Discrepancy Index (NADI). Pipeline implemented in Python (pandas, numpy, nltk).
Data source location	Ryan Research Institute (RRI), Paris, France
Data accessibility	Repository name: Zenodo Data identification number: 10.5281/zenodo.18680687 Direct URL to data: https://doi.org/10.5281/zenodo.18680687 Instructions for accessing these data: Open access; no login or embargo. Download the Parquet and CSV files directly from the Zenodo record page.
Related research article	None

## 1 Value of the Data

• ANAD v1 provides the first large-scale (*N* = 351,734), openly available derived feature resource for quantifying the discrepancy between narrative complexity and expressed affect at the population level.
• The dataset enables researchers in narrative psychology, clinical science, and computational social science to examine how structural elaboration and emotional expression co-vary in naturalistic personal narratives without requiring access to raw text.
• The transparent, deterministic annotation pipeline (LoC + VADER + NADI) allows direct replication and re-annotation with alternative models. Cross-corpus application requires recomputation of the LoC scaling on the target data.
• In the context of generative AI, the dataset serves as a human baseline against which model-generated narrative–affect patterns can be benchmarked.
• The signed discrepancy variant (NADI_signed) and sub-component features (loc_sent_var, loc_n_trans, loc_n_reinterp) support fine-grained analyses of directional misalignment and narrative micro-structure.
• The dataset opens concrete empirical questions—for instance, whether narrative–affect discrepancy predicts community engagement metrics such as comment volume, and whether this relationship differs across high-LoC and low-LoC regimes—that can be tested without access to raw text.

## 2 Background

Across psychology, psychiatry, and the humanities, human beings are often described as narrative agents whose sense of self is organized around constructions of past, present, and anticipated future experience [[2], [3], [4]]. Computational approaches have begun to map the large-scale emotional arcs of stories [5,6], yet these models track valence trajectories while discarding the structural agency of the narrator. Clinical research, conversely, has linked changes in narrative coherence and agency to trajectories of mental health [7,8], but rarely quantifies the affective surface against which that coherence operates. The result is a blind spot at the intersection of the two traditions: we know surprisingly little about when and how people's stories become structurally more complex than their expressed affect—as approximated by rule-based sentiment scoring—or, conversely, when intense emotion is carried by structurally simple narratives. This data article introduces the ANEST Narrative–Affect Dataset (ANAD v1) to address that gap. The theoretical development, construct validation, and psychological interpretation of the Narrative–Affect Discrepancy Index are presented in companion articles. The present article is restricted to documenting data collection procedures, the annotation pipeline, and reproducibility infrastructure.

## 3 Data Description

All data records are available from Zenodo under the persistent identifier https://doi.org/10.5281/zenodo.18680687 [14]. The current release (v1.3) contains three data files.

### 3.1 Primary data file

anad_canonical_v1.parquet (also available as anad_canonical_v1.csv) contains all *N* = 351,734 observations with 11 columns: id (platform-specific post identifier), word_count, sentence_count, text_length_char, LoC (0–10), sentiment_norm (0–10), NADI (|LoC − sentiment_norm|), NADI_signed (LoC − sentiment_norm), loc_sent_var, loc_n_trans, and loc_n_reinterp. This file contains no verbatim text, usernames, or personal metadata.

### 3.2 Supplementary data files

anad_mav_rms_only.parquet provides sentence-level VADER valence aggregations for the same *N* = 351,734 observations (columns: id, LoC, v_mean, v_mean_abs, v_mav, v_rms, v_sd, flip_rate).

loc_affect_corr_table.csv provides Pearson and Spearman correlations across LoC and affect variables.

### 3.3 Documentation and code

The full preprocessing and annotation pipeline is provided as a Jupyter notebook (anest_nadi_pipeline_v1.ipynb). Earlier versions of the Zenodo record contain additional documentation files, including a machine-readable schema, summary statistics, and a changelog; these can be accessed via the Zenodo version history.

Descriptive statistics for the main variables are summarized in Table 1.

**Table 1:** Descriptive statistics for key variables in ANAD v1.

Variable	N	M	SD
Word count	351,734	460.19	364.77
Sentence count	351,734	26.05	21.62
Character length	351,734	2372.81	1882.44
LoC (0–10)	351,734	2.44	1.81
Sentiment_norm (0–10)	351,734	6.50	4.16
NADI (0–10)	351,734	5.34	2.89

Table 2 maps the illustrative research questions from the Value of the Data section to the specific variables in the canonical file.

**Table 2:** Mapping of illustrative research questions to dataset variables.

Research question	Key variables
Does narrative complexity co-vary with expressed affect at the population level?	LoC, sentiment_norm, NADI
Is the narrative–affect discrepancy directional (over-narrated vs. under-narrated)?	NADI_signed
Which micro-structural features contribute most to narrative complexity?	loc_sent_var, loc_n_trans, loc_n_reinterp
Does narrative–affect discrepancy predict community engagement?	NADI, NADI_signed (linkable to external engagement data via id)
Can human narrative–affect patterns serve as a baseline for evaluating generative AI output?	All derived features

For illustration, a single observation in the canonical file takes the following form: id = ‘abc123’, word_count = 312, sentence_count = 18, text_length_char = 1740, LoC = 4.21, sentiment_norm = 6.85, NADI = 2.64, NADI_signed = −2.64, loc_sent_var = 22.7, loc_n_trans = 7, loc_n_reinterp = 2. All values shown are placeholders.

**Fig. 1:**
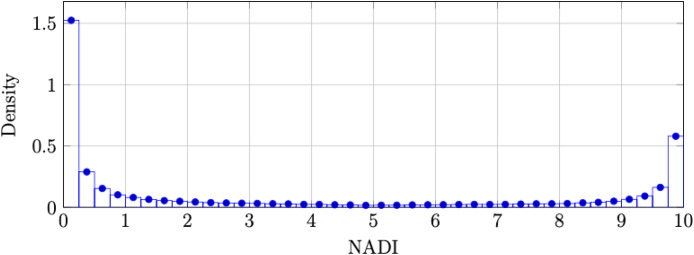
Empirical distribution of NADI across the corpus (*N* = 351,734).

**Fig. 2:**
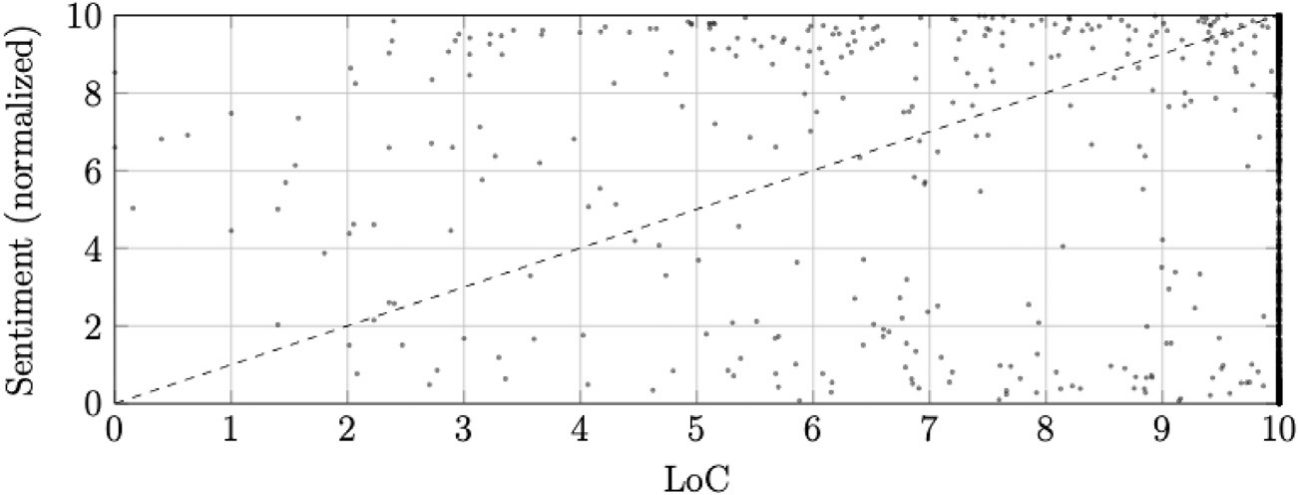
Relationship between LoC and sentiment_norm for a random subsample.

The empirical NADI distribution is shown in Fig. 1, and the joint distribution of LoC and sentiment_norm is shown in Fig. 2.

The histogram is binned into 40 intervals between 0 and 10.

Vertical distance from the dashed diagonal corresponds to NADI.

## 4 Experimental Design, Materials and Methods

### 4.1 Overall design

The construction of ANAD v1 proceeded in three stages. First, a large pool of candidate texts was assembled from publicly available online discussions in which individuals describe their own romantic or relational experiences in narrative form. Second, the raw texts were cleaned and filtered to remove extremely short, non-narrative, or malformed entries. Third, each remaining text was annotated with structural descriptors, a narrative complexity score (LoC), an affective polarity score, and the derived Narrative–Affect Discrepancy Index (NADI). All steps were implemented in Python using open-source libraries and are documented in the accompanying notebook.

### 4.2 Data source and sampling

ANAD v1 is built from publicly accessible posts in the subreddit r/relationships, where users discuss romantic and relational issues in a narrative format. Narratives were collected via the Pushshift API [1] and the official Reddit API over a multi-year window (2012–2023), then cleaned and processed at the Ryan Research Institute (RRI). Only a platform-specific post identifier and derived numerical features were retained for the released dataset; no Reddit usernames, profile information, or direct URLs are included at any stage.

As part of the provenance of the corpus, the pipeline also references an earlier public snapshot of the same subreddit hosted on Kaggle under the title “Reddit Relationships” [9], which includes the file reddit_small.csv containing post identifiers and text content. This Kaggle resource does not specify an exact temporal coverage window and is therefore treated here as an archival cross-sectional sample rather than as a full time series.

The final canonical dataset contains no duplicate post identifiers (*N* = 351,734 unique observations). Any potential overlap between the Pushshift/official API collection and the referenced Kaggle snapshot was resolved by deduplication on the platform-specific post identifier prior to feature extraction.

Because the corpus originates from Reddit's r/relationships community, it likely reflects platform-specific demographic and cultural biases in age, language use, and relationship norms that cannot be fully quantified in v1 due to the absence of user-level metadata.

### 4.3 Preprocessing and filtering

Preprocessing aimed to remove noise while preserving the semantic and affective content of the narratives. The following steps were applied:

1. **Removal of empty or trivial entries**. Rows with missing or whitespace-only content were dropped. Posts containing fewer than 10 whitespace-separated tokens were excluded to ensure minimal narrative coherence. The filtering was applied upstream during corpus assembly; the pre-filter pool was not archived separately, so the exact number of excluded entries cannot be reported. The released dataset (*N* = 351,734) reflects this filtered corpus and contains no entries with fewer than 10 tokens.
2. **Text normalization**. URLs were identified using a conservative regular expression and replaced with a single space. HTML tags, if present, were stripped. Line breaks were converted to spaces, and sequences of repeated whitespace were collapsed to a single space. No lemmatization, stemming, or lower-casing was applied at this stage; casefolding was deferred to the annotation functions where needed.
3. **Character and sentence counts**. For each cleaned text, the total number of characters and whitespace-separated tokens was computed. Sentences were approximated by splitting on punctuation marks (., !, ?), followed by trimming and removal of empty segments.

### 4.4 Level of complexity *(LoC)*

The Level of Complexity (LoC) is an interpretable heuristic index intended to capture aspects of narrative complexity that are not reducible to length alone. It combines sentence-level variability with the presence of discourse markers associated with temporal structure, contrast, and metacognitive reflection. Concretely, for each text:

1. The text is converted to lower case for pattern matching.
2. The text is segmented into sentences using a regular expression that splits on periods, exclamation marks, and question marks. Sentences with zero tokens are discarded.
3. The length (in tokens) of each sentence is computed, and the variance of sentence lengths is calculated.
4. Two lexicons of multiword patterns—motivated by the classical distinction between complicating action and evaluative clauses in narrative analysis [15] and by cognitive-processing word categories used in computational text analysis [6]—are counted:
  Transition markers: “but”, “however”, “although”, “then”, “after”, “before”, “when”, “while”, “so”, “therefore”, “because”, “yet”.
  Reinterpretation markers: “I thought”, “I realized”, “maybe”, “I guess”, “it felt like”, “seemed”, “apparently”.
5. A raw complexity score is computed as:
  LoC_raw = 0.1 × Var(sentence lengths) + (#transitions) + 1.5 × (#reinterpretations).
6. The raw scores are rescaled to the 0–10 range using a robust min–max transformation: the 1st and 99th percentiles of LoC_raw are mapped to 0 and 10, respectively, and values outside this interval are clipped.

The weight assignments reflect the following rationale. These weights were not derived from a manually labeled training subset; they are purely theory-driven, informed by the Labovian distinction between complicating action and evaluative clauses [15] and the cognitive-processing word-category framework used in computational text analysis [6]. Transition markers index the surface-level sequential progression of complicating action [15] and receive the baseline weight of 1.0. Sentence-length variance captures structural heterogeneity as a secondary indicator and is weighted at 0.1 to prevent it from dominating the composite. Reinterpretation markers signal evaluative and metacognitive reappraisal—a qualitatively distinct narrative operation that reorganizes the meaning of preceding events—and are weighted at 1.5 relative to transitions. The weights are not claimed to be optimal; they are provided to ensure transparent, reproducible baseline annotation. A brief sensitivity analysis varying the variance and reinterpretation weights across plausible ranges (0.05–0.20; 1.0–2.0) confirmed that LoC rankings remain highly stable (minimum Spearman ρ = 0.974), indicating that the specific weight values do not materially determine dataset structure. Because the 1st–99th percentile mapping compresses tail distributions, the rescaled LoC is a within-corpus relative index applied for interpretability on a common 0–10 scale with the sentiment component; it is not intended for cross-corpus metric comparison. Researchers working with other corpora should recompute the scaling using the provided pipeline.

### 4.5 Affective polarity and normalization

Affective polarity was estimated using VADER [10], a widely used rule-based sentiment analysis tool designed for social media text. VADER produces a compound score in the range [−1, 1] for each text. In the canonical dataset, the VADER compound score was computed on the full text of each narrative as a single input string (document-level scoring), not as an average of sentence-level scores. A separate supplementary file (anad_mav_rms_only.parquet) provides sentence-level VADER aggregations for researchers who require finer-grained affective profiling. VADER was selected for three reasons aligned with the goals of a reproducible data resource. First, as a fully deterministic, rule-based model with no external API or versioned model dependency, it ensures long-term reproducibility of the annotation layer. Second, sentence-level VADER compound scores can be aggregated to document level via standard summary statistics; the supplementary file anad_mav_rms_only.parquet provides such sentence-level aggregations (mean, mean absolute, moving average, RMS, SD, and flip rate) for all observations. Third, the deterministic nature of the tool means the released pipeline notebook can be adapted to substitute alternative affect estimators, including transformer-based models. The compound score was transformed to a 0–10 scale:

sentiment_norm = 5 × (compound + 1).

### 4.6 Narrative–affect discrepancy index (NADI)

The core derived variable is:

NADI = |LoC − sentiment_norm|.

A signed variant is also provided:

NADI_signed = LoC − sentiment_norm.

Because both components occupy the same bounded 0–10 scale, the absolute difference yields an interpretable discrepancy metric without requiring distributional assumptions or model fitting. Both absolute and signed variants are included in the canonical data file. A more advanced, residual-based variant of NADI—defined via generalized additive models—is introduced in a companion article and can be derived from ANAD v1 using the public pipeline.

### 4.7 Implementation and reproducibility

All preprocessing and annotation steps were implemented in Python using pandas, numpy, regex, and nltk. The full pipeline is provided as a Jupyter notebook (anest_nadi_pipeline_v1.ipynb) packaged together with the dataset on Zenodo (https://doi.org/10.5281/zenodo.18680687). The notebook is designed to run in a standard Google Colab environment without modification.

## Limitations

LoC is based on a small set of hand-crafted features with heuristic weights chosen for transparency rather than optimized against external criteria. It is a within-corpus relative index; cross-corpus comparisons should recompute the scaling using the provided pipeline. The absolute NADI definition does not distinguish “over-narrated” from “under-narrated” regimes; the signed variant addresses this. There is potential methodological entanglement between LoC and sentiment_norm, as some expressions that increase LoC can also attenuate VADER scores. For example, the phrase 'I thought everything was fine, but then I realized it was over' contains a transition marker ('but then') and a reinterpretation marker ('I realized'), which increase LoC, while the negative semantic content ('it was over') lowers the VADER compound score. In such cases, NADI may partly reflect shared lexical surface effects rather than a fully independent discrepancy between narrative structure and affect. Although the empirical correlation between the two is modest (*r* = 0.02), NADI should be treated as a transparent, computable text-based proxy rather than a validated measure of psychological dissociation.

The dataset represents a cross-sectional snapshot drawn from a single English-language online community (r/relationships). Findings derived from it may not generalize to other populations, languages, narrative genres, or temporal periods.

External construct validation of NADI against established clinical or psychometric instruments has not been performed in this release. The index is offered as a reproducible, computable indicator; its psychological interpretation is deferred to companion studies.

VADER was designed for short social-media text and has not been independently validated for long-form personal narratives. While the supplementary sentence-level aggregations mitigate this concern, comparison with transformer-based sentiment or dimensional emotion models remains a priority for future iterations.

The percentile-based rescaling (1st–99th percentile mapped to 0–10) compresses extreme values and produces a corpus-relative scale. Cross-corpus comparison of raw LoC values requires recomputation of the normalization on the target data. The canonical data file includes the sub-component columns (loc_sent_var, loc_n_trans, loc_n_reinterp), from which LoC_raw can be reconstructed as LoC_raw = 0.1 × loc_sent_var + loc_n_trans + 1.5 × loc_n_reinterp, enabling users to apply alternative normalization strategies.

Future iterations may replace the heuristic complexity layer with transformer-based estimators and the rule-based affect layer with dimensional emotion models, enabling the NADI framework to generalize beyond the lexical surface.

## Ethics Statement

The data were collected from a publicly accessible online discussion forum (social media platform). Participant data has been fully anonymized; only derived numerical features are released, and no individual can be re-identified from the dataset. No verbatim text, usernames, or personal metadata are redistributed. Original content can be reconstructed only via publicly available platform APIs using post identifiers, subject to the platform's terms of service. The platform's data redistribution policies were complied with by releasing derived features only. No re-identification of individual authors was attempted or is encouraged. The authors have read and follow the ethical requirements for publication in Data in Brief.

## CRediT Author Statement

**Ryan SangBaek Kim:** Conceptualization, Methodology, Software, Data curation, Formal analysis, Validation, Visualization, Writing – original draft, Writing – review & editing, Project administration.

## Data Availability

ZenodoANEST Narrative-Affect Representations (ANAD v1): Derived Feature Resource for Studying Narrative-Affect Discrepancy (Original data).

## Appendix Supplementary materials

Image, application 1
